# Anhaltende Fatigue als Folge einer COVID-19-Infektion bei Beschäftigten im Gesundheitswesen: Risikofaktoren und Auswirkungen auf die Lebensqualität

**DOI:** 10.1007/s00103-022-03511-4

**Published:** 2022-03-17

**Authors:** Julia Haller, Rüya-Daniela Kocalevent, Albert Nienhaus, Claudia Peters, Corinna Bergelt, Uwe Koch-Gromus

**Affiliations:** 1 Institut und Poliklinik für Medizinische Psychologie, Universitätsklinikum Hamburg-Eppendorf, Martinistr. 52, 20246 Hamburg, Deutschland; 2 Institut und Poliklinik für Allgemeinmedizin, Universitätsklinikum Hamburg-Eppendorf, Hamburg, Deutschland; 3 Institut für Versorgungsforschung in der Dermatologie und bei Pflegeberufen (IVDP), Universitätsklinikum Hamburg-Eppendorf, Hamburg, Deutschland; 4 Institut für Medizinische Psychologie, Universitätsmedizin Greifswald, Greifswald, Deutschland

**Keywords:** Postvirale Fatigue, Post-COVID, COVID-19 Survivors, Chronisches Fatigue-Syndrom, Rehabilitation, Post-viral fatigue, Post-COVID, COVID-19 survivors, Chronic fatigue syndrom, Rehabilitation

## Abstract

**Hintergrund und Ziel:**

Durch ihre Tätigkeit sind Menschen aus medizinisch-pflegerischen Berufen einem erhöhten Risiko für eine SARS-CoV-2-Infektion ausgesetzt und dadurch öfter von Krankheitsfolgen betroffen. In bisherigen Studien wurde als häufigste Krankheitsfolge die postvirale Fatigue (Erschöpfungssyndrom nach viraler Infektion) identifiziert. Das Ziel der Studie war die Untersuchung von Risikofaktoren für anhaltende Fatiguesymptome infolge einer COVID-19-Infektion und deren Auswirkungen bei Beschäftigten im Gesundheitswesen.

**Methoden:**

Im Frühjahr 2021 wurden 4315 Versicherte der Berufsgenossenschaft für Gesundheitsdienst und Wohlfahrtspflege (BGW) für eine schriftliche Befragung zu ihrer COVID-19-Erkrankung im Jahr 2020 und den Krankheitsfolgen angeschrieben. Dabei wurden Symptome der Akutinfektion, Krankheitsfolgen, mögliche Risikofaktoren sowie der körperliche und psychische Gesundheitszustand nach der SARS-CoV-2-Infektion erhoben. Als Fatiguescreening wurde die Skala „Allgemeine Erschöpfung“ des Multidimensional Fatigue Inventory (MFI) eingesetzt. Zur Datenanalyse wurden Regressionsanalysen und multivariate Varianzanalysen berechnet.

**Ergebnisse:**

10,7 % der Befragten wiesen schwere Fatiguewerte auf. Als Risikofaktoren für eine klinische Fatiguesymptomatik konnten u. a. Vorerkrankungen der Psyche und Atemwege sowie die Schwere der Akutinfektion identifiziert werden. Weiterhin war eine schwere Long‑/Post-COVID-Fatigue mit einer höheren psychischen Belastung, einer niedrigeren gesundheitsbezogenen Lebensqualität sowie mit einer häufigeren Arbeitsunfähigkeit assoziiert.

**Diskussion:**

Von schwerer Long‑/Post-COVID-Fatigue geht ein hoher Leidensdruck aus, der spezifische Rehabilitationsansätze erfordert und Sozialversicherungsträger sowie Unfallversicherer vor die Herausforderung stellt, geeignete Rehabilitationskonzepte zu entwickeln.

**Zusatzmaterial online:**

Zusätzliche Informationen sind in der Online-Version dieses Artikels (10.1007/s00103-022-03511-4) enthalten.

## Abstract (en)

**Background and objectives:**

Employees from medical and nursing professions are at increased risk for a SARS-CoV‑2 infection and thus more frequently affected by COVID-19 sequelae. Previous studies have identified post-viral fatigue as the most common sequelae. The aim of this study was to investigate risk factors and effects induced by clinically relevant fatigue symptoms following a COVID-19 infection of healthcare workers.

**Methods:**

In the spring of 2021, 4315 insured members of the Statutory Accident Insurance and Prevention in the Health and Welfare Service were contacted for a written survey on their COVID-19 disease in 2020 and its sequelae. Information on Symptoms of acute infection, disease sequelae, and potential risk factors were collected, as well as the physical and mental health status after SARS-CoV‑2 infection. The general fatigue scale of the Multidimensional Fatigue Inventory (MFI) was used as fatigue screening. Regression analyses and multivariate analyses of variance were calculated for data analysis.

**Results:**

Of the respondents, 10.7% showed severe fatigue symptoms. Identified risk factors for clinical fatigue symptoms included preexisting mental and respiratory conditions and severity of acute infection. Furthermore, severe long-/post-COVID fatigue was associated with higher psychological distress, lower health-related quality of life, and more frequent incapacity to work.

**Conclusions:**

Severe long-/post-COVID fatigue is associated with a high level of distress, which requires specific rehabilitation approaches and poses a challenge to the social insurance agencies and accident insurers to develop appropriate rehabilitation concepts.

## Einleitung

Das Coronavirus SARS CoV‑2 (Severe Acute Respiratory Syndrome Corona Virus Type 2) löste 2019 ein bis heute bestehendes weltweites Pandemiegeschehen aus. Dabei verursacht SARS-CoV‑2 die Erkrankung COVID-19, die verschiedene Organe und Systeme betreffen kann [1]. In der Bekämpfung der weltweiten Coronapandemie kommt Menschen aus medizinisch-pflegerischen Berufen eine zentrale Rolle zu. Gleichzeitig waren und sind Menschen aus diesen Berufsgruppen insbesondere bei Patient:innenkontakt einem sehr hohen Ansteckungsrisiko ausgesetzt [2, 3] und waren im Jahr 2020 besonders häufig von Arbeitsunfähigkeitszeiten wegen einer COVID-19-Diagnose betroffen [4]. Die Berufsgenossenschaft für Gesundheitsdienst und Wohlfahrtspflege (BGW) verzeichnete von Januar 2020 bis September 2021 insgesamt 118.566 meldepflichtige SARS-CoV-2-Fälle^1^ (Prof. Albert Nienhaus, persönliche Mitteilung, 30.09.2021). Die BGW ist der Unfallversicherungsträger für die private Kranken- und Wohlfahrtspflege einschließlich der Kirchen und anderer Nichtregierungsorganisationen. Entsprechend umfasst die BGW-Datenbank nicht die Beschäftigten in staatlichen Krankenhäusern [5].

Einige Betroffene leiden auch noch Wochen nach einer COVID-19-Erkrankung an anhaltenden oder neu auftretenden Beschwerden [6]. Während in der Literatur verschiedene Begriffe für diese Krankheitsfolgen verwendet werden (bspw. Long-COVID, Post-COVID, Long-Haulers, COVID sequelae), definiert die im Juli 2021 erschienene S1-Leitlinie [7] Beschwerden, die 4 Wochen nach Infektionsbeginn noch bestehen als „Long-COVID“. Eine Persistenz der Symptome über eine Dauer von 12 Wochen wird in der Leitlinie als „Post-COVID-Syndrom“ bezeichnet.

Daten aus bisherigen epidemiologischen Studien zeigen, dass die Mehrheit der Post-COVID-Betroffenen an einer Vielzahl von teilweise unspezifischen Symptomen leidet, darunter Müdigkeit, Atemnot, Geruchs- und Geschmacksstörungen, Schlafstörungen, Schmerzen in der Brust, Kopfschmerzen, Husten und psychische Probleme. Metaanalysen zur Häufigkeit der einzelnen Symptome des Post-COVID-Syndroms betonen eine große Heterogenität der jeweils eingeschlossenen Studien [6, 8, 9]. Generell legen bisherige Daten eine Abhängigkeit der Prävalenz von der untersuchten Bevölkerungsgruppe und deren Risikofaktoren nahe. Zwar weisen Risikoanalysen auf einen Zusammenhang zwischen der Schwere der Erkrankung und dem Auftreten von bestehenden Beschwerden hin [10], jedoch wird das Post-COVID-Syndrom auch bei milden COVID-19-Akutverläufen beobachtet [8]. Bisherige Studien zu Long‑/Post-COVID in der Allgemeinbevölkerung deuten auf massive Einschränkungen hinsichtlich Aktivität, Teilhabe und Lebensqualität hin [11]. Erste Ergebnisse zur Wirksamkeit von rehabilitativen Interventionen zur Behandlung von Spätsymptomen zeigen, dass Rehabilitationsmaßnahmen die Genesung nach einer SARS-CoV-2-Infektion beschleunigen können [12, 13].

Betroffene des Post-COVID-Syndroms berichten am häufigsten von Symptomen, die unter dem Begriff „Fatigue“ zusammengefasst werden [6]. Darunter fallen Symptome wie anhaltende Müdigkeit, eine verminderte Leistungsfähigkeit oder eine schnelle Erschöpfbarkeit (Post-Exertional Malaise). Häufig werden Fatiguesymptome auch von Kopf- und Gliederschmerzen und einer verminderten Konzentrationsfähigkeit begleitet. Entsprechend gilt das Vorliegen von Fatiguesymptomen als Risikofaktor für eine geringere Lebensqualität [9].

Anhaltende Fatiguesymptome sind dabei keine neuartige Erscheinung nach einer COVID-19-Infektion, sondern werden auch nach Infektionen mit anderen Viren, wie dem Epstein-Barr-Virus, dem humanen Herpesvirus oder Influenzaviren beobachtet und daher allgemein auch als „postvirales Fatiguesyndrom“ bezeichnet [14–16]. Ab einer Beschwerdedauer von 6 Monaten kann ein chronisches Fatigue-Syndrom (CFS; ICD-10: G93.3), auch „myalgische Enzephalomyelitis“ genannt, diagnostiziert werden. Trotz dieser Vorkenntnisse ist das Verständnis über Auftreten und Verlauf noch in den Anfängen. Dies betrifft auch die Einschränkungen in Aktivität und Teilhabe sowie die Rehabilitation bei Fatiguesymptomen als Teil des Post-COVID-Syndroms. Insbesondere fehlt es bislang an Daten zu Betroffenen aus Gesundheits- und Pflegeberufen in Deutschland, die durch ihre berufliche Tätigkeit besonderen Erkrankungsrisiken ausgesetzt sind.

Das Ziel dieser Studie ist es, Risikofaktoren für sowie Auswirkungen und Einschränkungen durch Fatiguesymptome als Folge einer COVID-19-Infektion von Beschäftigten im Gesundheitswesen zu untersuchen. Dabei soll analysiert werden, inwiefern sich Long‑/Post-COVID-Betroffene mit schweren, leichten oder fehlenden Fatiguesymptomen und Personen ohne Long‑/Post-COVID-Beschwerden hinsichtlich möglicher Risikofaktoren (bspw. Geschlecht, Gesundheitsverhalten, Vorerkrankungen), des Verlaufs der Akuterkrankung, der psychischen Belastung sowie der Lebensqualität unterscheiden. Dafür werden Daten aus einer Befragung von Versicherten der BGW, die im Jahr 2020 mit SARS-CoV‑2 infiziert waren, verwendet.

## Methoden

### Datenerhebung

Von Februar bis April 2021 wurden alle SARS-CoV-2-meldepflichtigen Fälle der Bezirksverwaltungen Köln und Dresden der BGW aus dem Jahr 2020 (*N* = 4315), davon 78 % weiblich, für eine schriftliche Befragung zur Nachverfolgung von Long‑/Post-COVID-Erkrankungsverläufen angeschrieben.

### Eingesetzte Instrumente

In dem Selbstauskunftsfragebogen wurden Symptome der Akutinfektion sowie weiterhin bestehende Symptome listenweise erfragt. Dabei wurden die Befragten gebeten sowohl auf sie zutreffende als auch nichtzutreffende Symptome entsprechend anzukreuzen. Fehlte die Angabe zu einem Symptom, wurde es bei der Auswertung als nicht vorhanden gewertet. Soziodemografische Daten inklusive des beruflichen Hintergrunds und des Erwerbsstatus wurden ebenso erhoben wie gesundheits- und versorgungsbezogene Daten zum allgemeinen Gesundheitsverhalten, zum subjektiven Gesundheitszustand *vor* und *nach* der COVID-19-Infektion, zu Vorerkrankungen, zur COVID-19-Infektion (Art der Testung und Testergebnis), Behandlung der Akuterkrankung, zur medizinischen Nachsorge und Rehabilitation sowie zur Leistungs- und Arbeitsfähigkeit vor und nach der COVID-19-Infektion.

Als Screeninginstrument für *Fatiguesymptome* wurde eine deutsche Übersetzung der Skala „Allgemeine Erschöpfung“ des Multidimensional Fatigue Inventory (MFI) verwendet. Das MFI ist ein 20 Items umfassendes Selbstauskunftsinstrument zur Messung von Fatigue und wurde in der englischen Version unter anderem an Patient:innen validiert, die an einem chronischen Fatiguesyndrom leiden [17, 18]. Die Skala „Allgemeine Erschöpfung“ umfasst 4 Items, die auf einer 5‑stufigen Likert-Skala (von „yes, that is true“ bis „no, that is not true“) beantwortet werden. Die Skala erfasst sowohl körperliche als auch psychologische Aspekte von Müdigkeit und Leistungsfähigkeit und wird daher auch als Screening in der Diagnostik von Fatiguesymptomen eingesetzt [19]. In der hier verwendeten deutschen Version werden die Items auf einer 11-stufigen Skala („0 = überhaupt nicht“ bis „10 = maximal“) beantwortet.

Die *gesundheitsbezogene Lebensqualität* wurde mit dem VR-12 (Veterans RAND 12-Item Health Survey) erhoben, der die beiden Subskalen *körperliche Summenskala* (PCS_90) und *psychische Summenskala* (MCS_90) beinhaltet [20]. Die interne Konsistenz der Summenskalen des VR-12 ist als akzeptabel bis gut zu bewerten. Höhere Werte in den Summenskalen entsprechen einer höher ausgeprägten körperlichen und psychischen Lebensqualität. Zur Erfassung von *Depressivität und Angst* wurde der PHQ‑4 (Patient Health Questionnaire for Depression and Anxiety-4) eingesetzt, der aus jeweils 2 Items zu Depressions- bzw. Angstsymptomen besteht [21]. Das Instrument weist eine hohe Reliabilität auf.

### Datenanalyse

In der Gesamtstichprobe wurden die zentralen statistischen Kennwerte der Skala „Allgemeine Erschöpfung“ des MFI erhoben und mit Daten einer Stichprobe gesunder Personen sowie einer Stichprobe von Personen mit CFS aus einer Validierungsstudie des MFI verglichen [18]. Für den Vergleich wurde die Skala „Allgemeine Erschöpfung“ entsprechend der englischsprachigen Originalskalierung transformiert (0–2 = 1, 3–4 = 2, 5–6 = 3, 7–8 = 4, 9–10 = 5).

Um mögliche Risikofaktoren zu untersuchen, die mit der späteren Entwicklung einer Long‑/Post-COVID-Fatigue zusammenhängen, wurde eine multiple Regressionsanalyse durchgeführt. In dieses Regressionsmodell wurden als Prädiktoren eingeschlossen: Geschlecht, Alter, berufliche Tätigkeit, Rauchen, Body-Mass-Index (BMI), der subjektive Gesundheitszustand vor der COVID-19-Infektion, Vorerkrankungen (Herz-Kreislauf-Erkrankungen, Atemwegserkrankungen, psychische Beeinträchtigung, Erkrankungen des Urogenitalsystems, Hauterkrankungen, Hormon‑/Stoffwechselerkrankungen), Symptome während der Akuterkrankung (Husten, Schnupfen, Halsschmerzen, Luftnot, Kopfschmerzen, Bauchschmerzen, Gliederschmerzen, Übelkeit, Durchfall, Fieber, Geruchs‑/Geschmacksstörungen, Müdigkeit) und Schwere der Akuterkrankung (Hospitalisierung, intensivmedizinische Behandlung, Beatmung). Die Variablenauswahl erfolgte durch Rückwärtsselektion.

Um weiterhin zu untersuchen, wie das Ausmaß der Long‑/Post-COVID-Fatigue mit dem Gesundheitszustand zum Befragungszeitpunkt assoziiert ist, wurde ein weiteres Regressionsmodell gerechnet, in das folgende unabhängige Variablen durch Rückwärtsselektion eingeschlossen wurden: subjektiver Gesundheitszustand nach der COVID-19-Infektion, psychische und körperliche gesundheitsbezogene Lebensqualität, psychische Belastung, Arbeitsunfähigkeit, Inanspruchnahme von Rehabilitationsmaßnahmen sowie der Wunsch nach Rehamaßnahmen. Aufgrund der Stichprobengröße wurde das Signifikanzniveau in beiden Regressionsanalysen auf α ≤ 0,01 gesetzt.

Zur Charakterisierung der Belastungen durch klinisch relevante Fatiguesymptome wurden 3 Subgruppen gebildet. Dafür wurde als klinischer Cut-off ein Wert von ≥ 16 im MFI angesehen, den auch Betroffene mit CFS in einer Validierungsstudie des MFI aufweisen [18]: 1. Versicherte mit klinisch relevanten Fatiguewerten; 2. Versicherte mit Fatiguewerten unter dem klinischen Cut-off, aber mindestens einem Long‑/Post-COVID-Symptom; 3. Versicherte ohne Long‑/Post-COVID-Beschwerden. Zum Vergleich der 3 Teilgruppen wurden Chi-Quadrat-Tests zur Analyse von Verteilungsunterschieden in kategorialen Merkmalen wie Vorerkrankungen, berufliche Tätigkeit etc. sowie Post-hoc-Tests mit Bonferroni-Korrektur durchgeführt.

Zuletzt wurde eine multivariate einfaktorielle Varianzanalyse (MANOVA) zur Untersuchung von Gruppenunterschieden in intervallskalierten Variablen wie Alter, subjektiver Gesundheitszustand vor und nach der COVID-19-Infektion etc. durchgeführt (α ≤ 0,01).

## Ergebnisse

### Gesamtstichprobe

Insgesamt nahmen *N* = 2052 Versicherte der BGW im Frühjahr 2021 an der Befragung zu ihrer COVID-19-Erkrankung im Jahr 2020 teil (Rücklaufquote: 47,6 %). Ausgeschlossen wurden alle Teilnehmenden, die nicht positiv auf SARS-CoV‑2 getestet worden waren oder dazu keine Angaben machten (*n* = 11). Weitere 6 Teilnehmende machten keine Angabe über ihre bestehende Long‑/Post-COVID-Symptomatik, sodass *n* = 2035 Personen in die finale Stichprobe eingeschlossen wurden. Davon waren 81,7 % weiblich, das Durchschnittsalter lag bei M = 47,86 (SD = 12,32) Jahren. Insgesamt *n* = 1180 (58,0 %) der Befragten gaben an, einer pflegerischen Tätigkeit nachzugehen, *n* = 197 (9,7 %) einer ärztlichen Tätigkeit, *n* = 116 (5,7 %) einer therapeutischen Tätigkeit, *n* = 78 (3,8 %) einer Verwaltungstätigkeit und *n* = 461 (22,7 %) einer anderen Tätigkeit. Nur 4,3 % der Teilnehmenden hatten zum Zeitpunkt der Befragung bereits eine Rehamaßnahme in Anspruch genommen, dagegen hätten sich 39,4 % eine Rehamaßnahme gewünscht.

### Analyse der Gesamtstichprobe

Die Gesamtstichprobe der befragten Versicherten gab im Mittel einen Fatiguewert auf der MFI-Skala von M = 11,19 (SD = 3,79) an. Dieser Wert ist verglichen mit der Stichprobe der gesunden Personen signifikant erhöht (M = 8,42, SD = 3,59; Cohen d = 0,735; 95 % KI [0,59, 0,88]), allerdings signifikant niedriger im Vergleich zu einer Stichprobe von Personen mit CFS (M = 16,38, SD = 2,73; Cohen d = −1,41; 95 % KI [−1,54, −1,28]; [18]).

Die Regressionsanalyse zur Vorhersage des Ausmaßes der Fatiguesymptomatik nach einer COVID-19-Infektion ergab, dass verschiedene Prädiktoren signifikant mit dem Ausmaß der Fatiguesymptomatik assoziiert sind (Tab. 1): weibliches Geschlecht, berufliche Tätigkeit, Rauchen, der subjektive Gesundheitszustand vor der COVID-19-Infektion, psychische Vorerkrankungen, Vorerkrankungen der Atemwege, die Anzahl der Akutsymptome, ein stationärer Aufenthalt während der COVID-19-Erkrankung sowie verschiedene Symptome während der Akuterkrankung (Husten, Schnupfen, Halsschmerzen, Luftnot, Kopfschmerzen, Bauchschmerzen, Gliederschmerzen, Übelkeit, Durchfall, Fieber, Geruchs‑/Geschmacksstörungen, Müdigkeit). Insgesamt erklärt das Modell 22,8 % der Varianz. Weiterhin war auch ein höherer BMI signifikant mit dem Ausmaß der Fatiguesymptomatik zum Befragungszeitpunkt assoziiert (r = 0,12, *p* < 0,001), wobei die Variable nicht in das Modell aufgenommen wurde, da sie keine zusätzliche Varianz erklärt. Nur geringfügig oder nicht signifikant assoziiert waren das Alter sowie andere Vorerkrankungen (Herz-Kreislauf-Erkrankungen, Erkrankungen des Urogenitalsystems, Hautkrankheiten, Hormon‑/Stoffwechselerkrankungen).

**Table 1:** 

Prädiktor	*R*^*a*^	*B*	*SE*	*β*	*t*	*p*
(Konstante)		15,47	1,14		13,62	< 0,001
*Weibliches Geschlecht*	0,15**	1,07	0,20	0,11	5,38	< 0,001
*Berufliche Tätigkeit*	0,06*	0,14	0,05	0,06	3,01	0,003
*Rauchen*	0,07**	0,75	0,21	0,07	3,59	< 0,001
*Psychische Vorerkrankungen*	0,15*	0,66	0,24	0,06	2,78	0,006
*Vorerkrankungen der Atemwege*	0,13*	0,67	0,24	0,06	2,86	0,004
*Subjektive Gesundheit vor COVID-19*	0,20**	−0,35	0,05	−0,14	−6,94	< 0,001
*Anzahl der Akutsymptome*	0,33**	1,91	0,19	1,72	10,11	< 0,001
*Akutsymptome*						
Husten	0,15**	−1,98	0,26	−0,25	−7,51	< 0,001
Schnupfen	0,10**	−1,96	0,26	−0,26	−7,61	< 0,001
Halsschmerzen	0,15**	−1,61	0,26	−0,21	−6,29	< 0,001
Luftnot	0,29*	−0,88	0,29	−0,12	3,05	0,002
Kopfschmerzen	0,20**	−1,67	0,29	−0,20	−5,80	< 0,001
Bauchschmerzen	0,18**	−1,69	0,30	−0,18	−5,63	< 0,001
Gliederschmerzen	0,19**	−2,04	0,30	−0,25	−6,79	< 0,001
Übelkeit/Erbrechen	0,22**	−1,26	0,30	−0,14	−4,25	< 0,001
Durchfall	0,17**	−1,74	0,28	−0,20	−6,29	< 0,001
Fieber	0,11**	−2,22	0,26	−0,29	−8,52	< 0,001
Geruchs‑/Geschmacksverlust	0,12**	−2,27	0,27	−0,29	−8,29	< 0,001
Müdigkeit/Erschöpfung	0,23**	1,39	0,36	−0,13	−3,91	< 0,001
*Stationäre Akutbehandlung*	0,10*	0,86	0,31	0,06	2,77	0,006

Anmerkungen: R^2^ = 0,228 (*n* = 1968, *p* < 0,01)

*p < 0,01, **p < 0,001

^a^Korrelation mit der Skala „Allgemeine Erschöpfung“ des multiplen Fatigue Inventory (MFI)

Die Regressionsanalyse zum Zusammenhang des Ausmaßes der Long‑/Post-COVID-Fatigue auf den Gesundheitszustand zum Befragungszeitpunkt ergab, dass der subjektive Gesundheitszustand nach der COVID-19-Infektion, die Arbeitsunfähigkeit sowie die psychische und körperliche gesundheitsbezogene Lebensqualität signifikant mit dem Ausmaß der Fatiguesymptomatik assoziiert sind (Tab. 2). Insgesamt erklärt dieses Modell 54 % der Varianz. Weiterhin waren die psychische Belastung (r = 0,54, *p* < 0,001), die Inanspruchnahme von Rehabilitationsleistungen (r = 0,06, *p* < 0,001) und der Wunsch nach Rehamaßnahmen (r = 0,38, *p* < 0,001) signifikant mit dem Ausmaß der Fatiguesymptomatik assoziiert, wobei diese Variablen nicht in das Modell aufgenommen wurden, da durch sie keine zusätzliche Varianz erklärt wurde.

**Table 2:** 

Prädiktor	*R*^*a*^	*B*	*SE*	*β*	*t*	*p*
(Konstante)		24,00	0,63		38,12	< 0,001
Subjektive Lebensqualität nach COVID-19	−0,66**	−0,56	0,05	−0,29	−11,29	< 0,001
Arbeitsfähigkeit	−0,18*	0,98	0,32	0,05	3,03	< 0,01
Körperliche gesundheitsbezogene Lebensqualität	−0,55**	−0,11	0,01	−0,33	−17,25	< 0,001
Psychische gesundheitsbezogene Lebensqualität	−0,57**	−0,12	0,01	−0,31	−13,65	< 0,001

Anmerkungen: R^2^ = 0,543 (*n* = 1772, *p* < 0,01)

*p < 0,01, **p < 0,001

^a^Korrelation mit der Skala „Allgemeine Erschöpfung“ des multiplen Fatigue Inventory (MFI)

### Analyse der Subgruppe von Betroffenen mit Long‑/Post-COVID-Fatigue

Insgesamt weisen 10,7 % (*n* = 217) der vorliegenden Stichprobe klinisch relevante Fatiguewerte (≥ 16) auf, die mit CFS-Betroffenen vergleichbar sind. Davon gaben 95,6 % an, seit der COVID-19-Infektion an diesen Symptomen zu leiden. 77,3 % der Stichprobe (*n* = 1574) gaben an, an Long‑/Post-COVID-Beschwerden zu leiden, wiesen jedoch Werte unterhalb des Cut-off auf. 12 % der Stichprobe (*n* = 244) gaben an, nicht an Long‑/Post-COVID-Beschwerden zu leiden. Tab. 3 gibt eine Übersicht über zentrale erhobene Kennwerte in den 3 Teilgruppen.

**Table 3:** 

	*LoPoCo-Fatigue (n* *=* *217)*	*LoPoCo-Sonstige (n* *=* *1574)*	*Non-LoPoCo (n* *=* *244)*	*Df*	*χ*^*2*^	*p*
	*n* (%)	*n* (%)	*n* (%)			
*Weibliches Geschlecht*	191 (88,0)	1296 (82,3)	176 (72,1)	2	*21,49*	< 0,001
*Alter in Jahren*	*M* = 47,59 (*SD* = 12,17)	*M* = 48,19 (*SD* = 12,14)	*M* = 45,96 (*SD* = 13,45)	–	*–*	*–*
*Body-Mass-Index (BMI)*	*M* = 27,68 (*SD* = 6,92)	*M* = 27,02 (*SD* = 5,82)	*M* = 26,17 (*SD* = 5,09)	–	*–*	*–*
*Raucher:innen*	41 (18,9)	244 (15,5)	41 (16,8)	2	1,80	0,41
*Körperliche Aktivität*				8	11,54	0,17
Keine körperliche Aktivität	72 (33,2)	457 (29,0)	80 (32,8)	–	–	–
1 h/Woche	52 (24,0)	380 (24,1)	51 (20,9)	–	–	–
2–3 h/Woche	56 (25,8)	464 (29,5)	61 (25,0)	–	–	–
> 3 h/Woche	29 (13,4)	246 (15,6)	46 (18,9)	–	–	–
*Vorerkrankungen im Bereich*				–	–	–
Herz/Kreislauf	62 (28,6)	419 (26,6)	41 (16,8)	2	14,37	< 0,05
Atemwege	39 (18,0)	194 (12,3)	16 (6,6)	2	9,50	< 0,01
Psyche	41 (18,9)	190 (12,1)	21 (8,6)	2	12,16	< 0,01
Urogenitalsystem	16 (7,4)	60 (3,8)	8 (3,3)	2	6,75	< 0,05
Haut	30 (13,8)	191 (12,1)	30 (12,3)	2	0,51	0,78
Hormone/Stoffwechsel	65 (30,0)	384 (24,4)	34 (13,9)	2	17,50	< 0,001
Andere	55 (25,3)	366 (23,3)	36 (14,8)	2	9,48	< 0,01
*Berufliche Tätigkeit*				2	23,94	< 0,01
Pflege	111 (51,2)	935 (59,4)	134 (54,9)	–	–	–
Ärztliche Tätigkeit	11 (5,1)	153 (9,7)	33 (13,5)	–	–	–
Therapeutische Tätigkeit	12 (5,5)	93 (5,9)	11 (4,5)	–	–	–
Verwaltungstätigkeit	13 (6,0)	57 (3,6)	8 (3,3)	–	–	–
Andere Tätigkeit	69 (31,8)	334 (21,2)	58 (23,8)	–	–	–
*Arbeitsunfähig seit COVID-19*	48 (22,1)	50 (3,2)	4 (1,6)	2	149,25	< 0,001
*Akutsymptome*				–	*–*	*–*
Anzahl der Akutsymptome	*M* = 8,53 (*SD* = 2,99)	*M* = 6,99 (*SD* = 3,34)	*M* = 4,98 (*SD* = 3,39)	–	–	*–*
Husten	159 (73,3)	990 (62,9)	116 (47,5)	2	32,23	< 0,001
Schnupfen	125 (57,6)	825 (52,4)	93 (38,1)	2	20,61	< 0,001
Halsschmerzen	146 (67,3)	843 (53,6)	105 (43,0)	2	27,68	< 0,001
Luftnot	176 (81,1)	887 (56,4)	76 (31,1)	2	115,34	< 0,001
Kopfschmerzen	179 (82,5)	1130 (71,8)	138 (56,6)	2	40,47	< 0,001
Bauchschmerzen	69 (31,8)	334 (21,2)	33 (13,5)	2	23,46	< 0,001
Gliederschmerzen	171 (78,8)	1103 (70,1)	120 (49,2)	2	56,11	< 0,001
Übelkeit/Erbrechen	79 (36,4)	333 (21,9)	25 (10,2)	2	47,58	< 0,001
Durchfall	89 (41,0)	417 (26,5)	47 (19,3)	2	29,50	< 0,001
Fieber	123 (56,7)	802 (51,0)	98 (40,2)	2	12,60	< 0,01
Geruchs‑/Geschmacksverlust	152 (70,0)	1022 (64,9)	123 (50,4)	2	24,01	< 0,001
Müdigkeit/Erschöpfung	207 (95,4)	1360 (86,4)	169 (69,3)	2	67,30	< 0,001
Konzentrations‑/Gedächtnisstörungen	177 (81,6)	943 (59,9)	71 (29,1)	2	136,36	< 0,001
*Stationäre Behandlung der Akuterkrankung*	30 (13,8)	99 (6,3)	6 (2,5)	2	25,41	< 0,001
*Intensivmedizinische Behandlung*	7 (3,2)	28 (1,8)	–	2	7,34	< 0,05
*Subjektiver Gesundheitszustand vor COVID-19*	*M* = 9,73 (*SD* = 1,38)	*M* = 10,12 (*SD* = 1,18)	*M* = 10,29 (*SD* = 0,98)	–	*–*	*–*
*Subjektiver Gesundheitszustand nach COVID-19*	*M* = 5,63 (*SD* = 2,06)	*M* = 8,50 (*SD* = 1,75)	*M* = 9,99 (*SD* = 1,17)	–	*–*	*–*
*Long‑/Post-COVID-Symptome*				–	–	–
Husten	49 (22,6)	275 (17,5)	–	2	56,72	< 0,001
Schnupfen	28 (12,9)	169 (10,7)	–	2	31,01	< 0,001
Halsschmerzen	19 (8,8)	133 (8,4)	–	2	23,03	< 0,001
Luftnot	118 (54,4)	737 (46,8)	–	2	207,41	< 0,001
Kopfschmerzen	85 (39,2)	547 (34,8)	–	2	127,68	< 0,001
Bauchschmerzen	14 (6,5)	119 (7,6)	–	2	20,09	< 0,001
Gliederschmerzen	69 (31,8)	444 (28,2)	–	2	95,81	< 0,001
Übelkeit/Erbrechen	11 (5,1)	84 (5,3)	–	2	13,87	< 0,01
Durchfall	18 (8,3)	87 (5,5)	–	2	18,25	< 0,001
Fieber	5 (2,3)	22 (1,4)	–	2	4,98	0,08
Geruchs‑/Geschmacksverlust	66 (30,4)	517 (32,8)	–	2	113,31	< 0,001
Müdigkeit/Erschöpfung	157 (72,4)	1097 (69,7)	–	2	449,97	< 0,001
Konzentrations‑/Gedächtnisstörungen	138 (63,6)	929 (59,0)	–	2	310,20	< 0,001
Andere Symptome	71 (32,7)	306 (19,4)	–	2	86,06	< 0,001
*Psychische Belastung (PHQ-4)*	*M* = 5,39 (*SD* = 2,97)	*M* = 2,63 (*SD* = 3,46)	*M* = 1,55 (*SD* = 2,00)	–	*–*	*–*
*Psychische Summenskala VR-12*	*M* = 33,54 (*SD* = 9,97)	*M* = 44,28 (*SD* = 8,90)	*M* = 51,29 (*SD* = 6,86)	–	*–*	*–*
*Körperliche Summenskala VR-12*	*M* = 34,12 (*SD* = 10,97)	*M* = 45,68 (*SD* = 11,30)	*M* = 50,89 (*SD* = 9,93)	–	*–*	*–*
*Ambulante Nachbetreuung*	109 (48,0)	493 (31,3)	23 (9,4)	2	90,64	< 0,001
Allgemeinmedizin	89 (41,0)	401 (25,5)	18 (7,4)	2	71,96	< 0,001
Kardiologie	34 (15,7)	107 (6,8)	3 (1,2)	2	38,07	< 0,001
Pulmologie	43 (19,8)	143 (9,1)	4 (1,6)	2	46,45	< 0,001
Neurologie	19 (8,8)	26 (1,7)	0 (0)	2	50,72	< 0,001
Andere	26 (12,0)	84 (5,3)	4 (1,6)	2	24,67	< 0,001
*Stationäre Rehamaßnahme*	18 (8,3)	38 (2,4)	–	2	31,84	< 0,001
*Wunsch nach Rehabilitation*	135 (72,97)	557 (39,14)	29 (11,87)	2	156,94	< 0,001

*PHQ‑4* Patient Health Questionnaire for Depression and Anxiety‑4, *VR-12* Veterans RAND 12-Item Health Survey

In der Teilgruppe der von schwerer Fatigue Betroffenen sind 88,0 % weiblich, das Durchschnittsalter beträgt M = 47,59 (SD = 12,17) Jahre. Die Chi-Quadrat-Tests weisen auf Unterschiede zwischen den Häufigkeiten der meisten untersuchten Variablen bei den 3 Teilgruppen hin. Keine Unterschiede zwischen den Teilgruppen wurden lediglich für das Rauchverhalten, die körperliche Aktivität, Vorerkrankungen im Bereich der Haut und für Fieber als Long‑/Post-COVID-Symptom gefunden (vgl. Tab. 3).

Post-hoc-Tests mit Bonferroni-Korrektur zeigen für die Fatigueteilgruppe signifikant häufiger Vorerkrankungen im Bereich der Psyche (z = 3,1) und der Atemwege (z = 2,7), ein häufigeres Vorkommen der meisten Akutsymptome (Husten: z = 3,6; Halsschmerzen: z = 4,3; Luftnot: z = 7,9; Kopfschmerzen: z = 3,9; Bauchschmerzen: z = 3,9; Übelkeit/Erbrechen: z = 5,4; Durchfall: z = 4,8; Müdigkeit: z = 44; Konzentrations- und Gedächtnisprobleme: z = 7,3; andere Symptome: z = 3,8) sowie häufigere stationäre Behandlungen (z = 4,5) als die beiden andere Teilgruppen auf. Ebenso wies die von schwerer Fatigue betroffene Teilgruppe signifikant häufiger die Long‑/Post-COVID-Symptome Husten (z = 2,8), Luftnot (z = 3,9), Kopfschmerzen (z = 2,7), Müdigkeit (z = 3,4), Konzentrations- und Gedächtnisprobleme (z = 3,5) sowie andere Symptome (z = 5,7) auf und war häufiger arbeitsunfähig (z = 12,2) als die beiden anderen Teilgruppen. Auch nahm die von schwerer Fatigue betroffene Teilgruppe signifikant häufiger eine ambulante medizinische Nachbetreuung (z = 6,5) sowie stationäre Rehamaßnahmen in Anspruch (z = 5,2) und gab häufiger den Wunsch nach einer Rehabilitationsmaßnahme an (z = 9,9).

Der Gruppenvergleich mittels multivariater Varianzanalyse zeigt, dass die 3 Teilgruppen sich systematisch unterscheiden im Hinblick auf den subjektiven Gesundheitszustand vor und nach der COVID-19-Erkrankung, die Anzahl der Akutsymptome, die psychische Belastung sowie die psychische und körperliche gesundheitsbezogene Lebensqualität (*p* < 0,001; s. Onlinematerial).

Die Post-hoc-Analysen zeigen, dass sich alle 3 Teilgruppen in der Wahrnehmung der subjektiven Gesundheit nach der Infektion sowie in der Anzahl der Akutsymptome, der psychischen Belastung und hinsichtlich der psychischen und körperlichen gesundheitsbezogenen Lebensqualität unterscheiden, wobei die Teilgruppe der von schwerer Fatigue Betroffenen die höchsten gesundheitlichen Belastungen zeigte und die Teilgruppe der Long‑/Post-COVID-Betroffenen mit Fatiguewerten unter dem klinischen Cut-off die zweithöchste Belastung (Tab. 4). Dagegen weist die Teilgruppe der von schwerer Fatigue Betroffenen im Hinblick auf die Wahrnehmung der subjektiven Gesundheit vor der Infektion eine höhere Belastung auf als die anderen beiden Teilgruppen, während Letztere sich nicht unterscheiden.

**Table 4:** 

Paarweiser Vergleich	*M*_*Diff*_	*SE*	*p*	*95* *% KI*	
				*UG*	*OG*
*Subjektiver Gesundheitszustand vor COVID-19*					
LoPoCo-Fatigue vs. LoPoCo-Sonstige	−0,40	0,10	< 0,01	−0,64	−0,16
LoPoCo-Fatigue vs. non-LoPoCo	−0,55	0,12	< 0,001	−0,82	−0,28
LoPoCo-Sonstige vs. non-LoPoCo	−0,15	0,07	0,093	−0,32	0,02
*Subjektiver Gesundheitszustand nach COVID-19*					
LoPoCo-Fatigue vs. LoPoCo-Sonstige	−2,79	0,15	< 0,001	−3,15	−2,43
LoPoCo-Fatigue vs. non-LoPoCo	−4,28	0,16	< 0,001	−4,67	−3,90
LoPoCo-Sonstige vs. non-LoPoCo	−1,49	0,09	< 0,001	−1,70	−1,29
*Anzahl der Akutsymptome*					
LoPoCo-Fatigue vs. LoPoCo-Sonstige	1,60	0,23	< 0,001	1,06	2,13
LoPoCo-Fatigue vs. non-LoPoCo	3,65	0,31	< 0,001	2,93	4,37
LoPoCo-Sonstige vs. non-LoPoCo	2,05	0,24	< 0,001	1,49	2,61
*Psychische Belastung (PHQ-4)*					
LoPoCo-Fatigue vs. LoPoCo-Sonstige	2,70	0,22	< 0,001	2,18	3,22
LoPoCo-Fatigue vs. non-LoPoCo	3,84	0,25	< 0,001	3,25	4,42
LoPoCo-Sonstige vs. non-LoPoCo	1,14	0,15	< 0,001	0,79	1,48
*Gesundheitsbezogene Lebensqualität: psychische Summenskala VR-12*					
LoPoCo-Fatigue vs. LoPoCo-Sonstige	−10,50	0,74	< 0,001	−12,24	−8,75
LoPoCo-Fatigue vs. non-LoPoCo	−17,48	−0,83	< 0,001	−19,44	−15,52
LoPoCo-Sonstige vs. non-LoPoCo	−6,99	0,50	< 0,001	−8,17	−5,81
*Gesundheitsbezogene Lebensqualität: körperliche Summenskala VR-12*					
LoPoCo-Fatigue vs. LoPoCo-Sonstige	−11,72	0,83	< 0,001	−13,69	−9,76
LoPoCo-Fatigue vs. non-LoPoCo	−16,83	1,02	< 0,001	−19,22	−14,43
LoPoCo-Sonstige vs. non-LoPoCo	−5,10	0,72	< 0,001	−6,79	−3,42

Anmerkung: Aufgrund fehlender Werte in einer oder mehreren untersuchten Variablen wurden *n* = 98 Teilnehmende aus der Analyse ausgeschlossen. Eingeschlossen wurden aus der Teilstichprobe LoPoCo-Fatigue: *n* = 197, aus der Teilstichprobe LoPoCo-Sonstige: *n* = 1505 und aus der Teilstichprobe non-LoPoCo: *n* = 235

*PHQ‑4* Patient Health Questionnaire for Depression and Anxiety‑4, *VR-12* Veterans RAND 12-Item Health Survey

## Diskussion

Unsere Studie untersuchte die Risikofaktoren für und gesundheitlichen Auswirkungen von schwerer Long‑/Post-COVID-Fatigue von Beschäftigten in Gesundheitsberufen in Deutschland anhand einer Befragung von BGW-Versicherten, die 2020 mit SARS-CoV‑2 infiziert waren. Unter allen von Long‑/Post-COVID-19 Betroffenen stellen diejenigen, die an schweren Fatiguesymptomen leiden, eine besonders vulnerable Gruppe dar. So weisen die Ergebnisse einerseits darauf hin, dass die Fatiguesymptomatik insgesamt unter Long‑/Post-COVID-19-Betroffenen signifikant höher ausgeprägt ist als in der Allgemeinbevölkerung [18]. Andererseits verdeutlichen sowohl die Regressionsanalyse zu den Auswirkungen von Fatigue als auch die Ergebnisse aus dem Vergleich der Teilgruppen, dass eine schwere Long‑/Post-COVID-Fatigue mit einer höheren psychischen Belastung, stärkeren Einschränkungen hinsichtlich der gesundheitsbezogenen Lebensqualität, einem niedrigeren subjektiven Gesundheitszustand sowie mit einer häufigeren Arbeitsunfähigkeit assoziiert ist. Damit stimmen unsere Ergebnisse mit Erkenntnissen aus einer kürzlich erschienenen Metaanalyse von Malik und Kolleg:innen [9] überein, in der Long‑/Post-COVID-Fatigue mit einer niedrigeren Lebensqualität assoziiert war.

Gleichzeitig zeigte sich in unseren Analysen, dass Menschen, die von schwerer Long‑/Post-COVID-Fatigue betroffen sind, unter einem besonderen Leidensdruck stehen, wenn die Schwere der Symptomatik mit der von CFS-Erkrankten vergleichbar ist. Dies spiegelte sich auch in der häufigeren Inanspruchnahme ambulanter medizinischer Nachbehandlungen und stationärer Rehabilitationsmaßnahmen sowie in dem häufigen Wunsch nach Rehabilitationsangeboten der untersuchten Teilgruppe wider.

Für die Entwicklung einer schweren Fatiguesymptomatik konnten sowohl in der Regressionsanalyse der Risikofaktoren als auch in den Analysen der Teilgruppen folgende Risikofaktoren identifiziert werden: weibliches Geschlecht, ein schlechterer subjektiver Gesundheitszustand vor der COVID-19-Infektion, psychische Vorerkrankungen, Vorerkrankungen der Atemwege sowie Schwere der Akutinfektion (Anzahl der Akutsymptome, Hospitalisierung). Dagegen schienen das Alter, Herz-Kreislauf-Erkrankungen, Erkrankungen des Urogenitalsystems oder der Haut, Hormon- und Stoffwechselerkrankungen das Risiko für ein Long‑/Post-COVID-Syndrom mit schwerer Fatigue nicht zu erhöhen. Ebenso konnte für geringe körperliche Aktivität und Höhe des BMI kein erhöhtes Risiko für einen Long‑/Post-COVID-Krankheitsverlauf mit schwerer Fatigue identifiziert werden. Auch die Faktoren berufliche Tätigkeit und Rauchen zeigten nur sehr geringe Zusammenhänge mit dem Ausmaß der Fatigue und es zeigten sich keine Unterschiede zwischen den Teilgruppen mit schwerer, leichter oder ohne Long‑/Post-COVID-Symptomatik.

### Implikationen für die Versorgung und zukünftige Forschung

In unserer Stichprobe wurde bei 10,7 % der Befragten eine schwere Symptomatik identifiziert, die mit jener von CFS-Betroffenen vergleichbar ist. Rund 73 % der Befragten äußerten einen Rehabedarf. Doch auch bei moderaten Fatiguewerten ist von einer klinischen Relevanz auszugehen. So litten weitere 77,3 % unter milderen Fatiguesymptomen oder anderen Long‑/Post-COVID-Symptomen. Auch in dieser Gruppe gaben fast 40 % einen Rehabedarf an.

Bereits heute stellt der hohe Leidensdruck der Betroffenen eine besondere Herausforderung für die Rehabilitation dar. Zwar bestätigen Studien die Wirksamkeit stationärer Rehabilitationsmaßnahmen, jedoch fehlt es bislang an Programmen, die spezifisch auf die Bedürfnisse von Betroffenen mit schwerer Fatiguesymptomatik ausgerichtet sind. In zukünftigen Studien sollten daher die Behandlungsbedürfnisse von Fatiguebetroffenen allgemein und spezifisch von Beschäftigten im Gesundheitswesen multiperspektivisch untersucht werden, um Rehabilitationsansätze entsprechend zu adaptieren.

Aufgrund der Neuartigkeit von SARS-CoV‑2 fehlt es bislang an Studien, die den Verlauf von Post-COVID-Fatigue prospektiv untersuchen. Erfahrungen zu Langzeitverläufen von postviraler Fatigue bei anderen Infektionskrankheiten deuten auf die Gefahr einer Chronifizierung der Symptomatik hin [15]. Für die Prävention von schwerer Fatigue infolge einer SARS-CoV-2-Infektion könnte Impfungen eine herausragende Bedeutung zukommen. Denn neben der Senkung des Ansteckungsrisikos wird durch eine Impfung gegen SARS-CoV‑2 auch die Wahrscheinlichkeit eines schweren Krankheitsverlaufs und damit auch für die Entwicklung einer postviralen Fatigue entscheidend gesenkt [22]. In zukünftigen Studien sollte dementsprechend die Wirkung der verschiedenen COVID-19-Impfstoffe zur Prävention von Fatigue und anderen Long‑/Post-COVID-Beschwerden untersucht werden.

### Stärken und Limitationen

Unsere Studie stellt nach unserem Kenntnisstand die erste Analyse von Risikofaktoren für die Entwicklung einer schweren Long‑/Post-COVID-Fatigue (auf dem Niveau von CFS-Betroffenen) und für deren gesundheitliche Auswirkungen dar. Dafür wurden in unserer Studie Long‑/Post-COVID-Betroffene nicht einheitlich betrachtet, sondern nach der Schwere der Fatiguesymptomatik differenziert. Während in verschiedenen Metaanalysen die Prävalenz von Long‑/Post-COVID-Fatigue zwischen 35–60 % geschätzt wird [6, 8], deuten unsere Ergebnisse darauf hin, dass der Anteil von Betroffenen mit schwerer Fatigue deutlich geringer ist.

Die Differenzierung der Stichprobe ermöglicht eine genauere Analyse der Therapie- und Rehabilitationsbedürfnisse von Long‑/Post-COVID-Betroffenen. Dabei wurde mit den BGW-Versicherten eine besonders von der Pandemie betroffene Zielgruppe untersucht. Durch den direkten Zugang über die Unfallversicherung BGW konnten alle SARS-CoV-2-meldepflichtigen Fälle aus 2 Bezirksverwaltungen angesprochen werden, sodass von einer hohen Generalisierbarkeit bei der Rekrutierung der Stichprobe ausgegangen werden kann. Trotz der hohen Rücklaufquote (48 %) ist jedoch von einem selektiven Antwortverhalten von Long‑/Post-COVID-Betroffenen auszugehen.

Einschränkend zu erwähnen ist zudem die subjektive und teilweise retrospektive Einschätzung einiger erhobener Variablen (bspw. subjektiver Gesundheitszustand *vor* der COVID-19-Infektion), wodurch systematische Fehler im Antwortverhalten (bspw. Erinnerungseffekte) begünstigt werden können. Die Einschätzung der Fatiguesymptomatik erfolgte anhand der Screeningskala „Allgemeine Erschöpfung“ des MFI und stellt somit keine gesicherte Diagnostik von CFS dar. Weiterhin erschwert die in dieser Studie verwendete 11-stufige Skalierung den Vergleich der Ergebnisse mit Normwerten des ursprünglich 5‑stufigen MFI und mit internationalen Publikationen.

## Fazit

Von schwerer Long‑/Post-COVID-Fatigue Betroffene sind starken psychischen und körperlichen Belastungen ausgesetzt. Gleichzeitig äußern diese einen hohen Rehabedarf, den es in der Planung von Rehabilitationsprogrammen zu berücksichtigen gilt.

## Supplementary Information





## Footnotes

1 Von einem meldepflichtigen Fall wird dann gesprochen, wenn ein begründeter Verdacht auf eine COVID-19-Erkrankung durch PCR-Test oder Symptome sowie begründeter Verdacht auf beruflichen Zusammenhang und fehlenden Hinweis auf private Ursache bestand. Versicherungsfälle, die im Jahr 2020 erkrankt, aber bis Februar 2021 nicht gemeldet wurden (= Nachmeldungen), wurden nicht mitberücksichtigt.
