# Supplementary material for: Persistent fatigue symptoms following COVID-19 infection in healthcare workers: risk factors and impact on quality of life
Source: Bundesgesundheitsblatt Gesundheitsforschung Gesundheitsschutz. 2022 Mar 17;65(4):471–80. [Article in German] doi: 10.1007/s00103-022-03511-4 (PMC8928711; doi:10.1007/s00103-022-03511-4)
Supplement: Supplementary file 1 [file 103_2022_3511_MOESM1_ESM.pdf]

Bundesgesundheitsblatt – Gesundheitsforschung – Gesundheitsschutz, Ausgabe 4/2022

Onlinematerial zum Beitrag:

Anhaltende Fatigue als Folge einer COVID-19-Infektion bei Beschäftigten im Gesundheitswesen: Risikofaktoren und Auswirkungen auf die Lebensqualität

Julia Haller[1*], Rüya-Daniela Kocalevent[2], Albert Nienhaus[3], Claudia Peters[3], Corinna Bergelt[4**], Uwe Koch-Gromus[1**]

[1] Universitätsklinikum Hamburg-Eppendorf, Institut und Poliklinik für Medizinische Psychologie, Hamburg, Deutschland
[2] Universitätsklinikum Hamburg-Eppendorf, Institut und Poliklinik für Allgemeinmedizin, Hamburg, Deutschland
[3] Universitätsklinikum Hamburg-Eppendorf, Institut für Versorgungsforschung in der Dermatologie und bei Pflegeberufen (IVDP), Hamburg, Deutschland
[4] Universitätsmedizin Greifswald, Institut für Medizinische Psychologie, Greifswald, Deutschland

[*]Korrespondierende Autorin
[**]geteilte Letztautorenschaft (beide Autor:innen haben gleichermaßen zum Manuskript beigetragen)

Korrespondenzadresse:
Julia Haller-Wolf
Universitätsklinikum Hamburg-Eppendorf
Institut und Poliklinik für Medizinische Psychologie
Martinistraße 52
20246 Hamburg
Deutschland
j.haller-wolf@uke.de

Inhalt:

Tabelle: Vergleich der Versicherten der Berufsgenossenschaft für Gesundheitsdienst und Wohlfahrtpflege (BGW), die 2020 mit SARS-CoV-2 infiziert waren, unterteilt in drei Teilgruppen: von schwerer Long-/Post-COVID-19-Fatigue Betroffene (LoPoCoFatigue); von leichter Fatigue Betroffene (LoPoCo-Sonstige) bzw. nicht von Fatigue Betroffene (non-LoPoCo) mittels multivariater Varianzanalyse

Vergleich der Versicherten der Berufsgenossenschaft für Gesundheitsdienst und Wohlfahrtpflege (BGW), die 2020 mit SARS-CoV-2 infiziert waren, unterteilt in drei Teilgruppen: von schwerer Long-/Post-COVID-19-(LoPoCo-)Fatigue Betroffene (LoPoCoFatigue); von leichter LoPoCo-Fatigue Betroffene (LoPoCo-Sonstige) bzw. nicht von LoPoCo-Fatigue Betroffene (non-LoPoCo) mittels multivariater Varianzanalyse

	df1	*df2*	*F*	*p*	η^2_{part}
Alter	2	1937	3.52	0,030	0,004
Body-Mass-Index (BMI)	2	1937	3.87	0,021	0,004
subjektiver Gesundheitszustand vor COVID-19	2	1937	13.26	<0,001	0,011
subjektiver Gesundheitszustand nach COVID-19	2	1937	348.55	<0,001	0,331
Anzahl der Akutsymptome	2	1937	68.25	<0,001	0,152
PHQ-4	2	1937	142.79	<0,001	0,099
Psychische Summenskala VR-12	2	1937	215.66	<0,001	0,279
Körperliche Summenskala VR-12	2	1937	133.33	<0,001	0,150

Anmerkung: Aufgrund fehlender Werte in einer oder mehreren untersuchten Variablen wurden *n*= 98 Teilnehmende aus der Analyse ausgeschlossen, sodass aus der Teilstichprobe LoPoCo-Fatigue *n*= 197, aus der Teilstichprobe LoPoCo-Sonstige *n*= 1.505 und aus der Teilstichprobe non-LoPoCo *n*= 235 Teilnehmende in die Analyse eingeschlossen wurden.

PHQ-4 = Patient Health Questionnaire for Depression and Anxiety-4, VR-12= Veterans RAND 12-Item Health Survey
